# Detecting the pulmonary trunk in CT scout views using deep learning

**DOI:** 10.1038/s41598-021-89647-w

**Published:** 2021-05-13

**Authors:** Aydin Demircioğlu, Magdalena Charis Stein, Moon-Sung Kim, Henrike Geske, Anton S. Quinsten, Sebastian Blex, Lale Umutlu, Kai Nassenstein

**Affiliations:** 1 Department of Diagnostic and Interventional Radiology and Neuroradiology, University Hospital Essen, University of Duisburg-Essen, Hufelandstr. 55, 45147 Essen, Germany; 2 Department of Surgery and Orthopedics, Landesspital Liechtenstein, Heiligkreuz 25, 9490 Vaduz, Liechtenstein

**Keywords:** Computed tomography, Machine learning, Data acquisition

## Abstract

For CT pulmonary angiograms, a scout view obtained in anterior–posterior projection is usually used for planning. For bolus tracking the radiographer manually locates a position in the CT scout view where the pulmonary trunk will be visible in an axial CT pre-scan. We automate the task of localizing the pulmonary trunk in CT scout views by deep learning methods. In 620 eligible CT scout views of 563 patients between March 2003 and February 2020 the region of the pulmonary trunk as well as an optimal slice (“reference standard”) for bolus tracking, in which the pulmonary trunk was clearly visible, was annotated and used to train a U-Net predicting the region of the pulmonary trunk in the CT scout view. The networks’ performance was subsequently evaluated on 239 CT scout views from 213 patients and was compared with the annotations of three radiographers. The network was able to localize the region of the pulmonary trunk with high accuracy, yielding an accuracy of 97.5% of localizing a slice in the region of the pulmonary trunk on the validation cohort. On average, the selected position had a distance of 5.3 mm from the reference standard. Compared to radiographers, using a non-inferiority test (one-sided, paired Wilcoxon rank-sum test) the network performed as well as each radiographer (P < 0.001 in all cases). Automated localization of the region of the pulmonary trunk in CT scout views is possible with high accuracy and is non-inferior to three radiographers.

## Introduction

Pulmonary embolism (PE) is the third leading cause of cardiovascular death with a high rate of unreported cases^1^. The annual incidence of PE is approximately 600 to 2.000 cases/million^2^ and increases significantly with age, exceeding 5.000 cases/million in those over 70 years of age^3^.

Computed tomography of the pulmonary arteries (CTPA) is recommended as first-line imaging modality in the setting of suspected PE because of its high diagnostic accuracy^4,5^. Unfortunately, CTPA has two decisive method-related disadvantages: First, radiation exposure and second, the need for intravenous contrast media injection. Due to the risk of contrast media induced nephropathy, an intravenous administration of contrast media is problematic in patients with impaired renal function^6–8^. Unfortunately, many patients with suspected PE are elderly and therefore have impaired renal function, which practically means that the smallest possible amount of contrast media should be used in these patients.

The basic requirement for a meaningful CTPA, especially when using low amounts of contrast media, is an optimal timing of the CT acquisition, which means that the scan must be acquired at the moment when the pulmonary arteries are optimally contrasted. Bolus tracking is the most widespread method used to achieve this. With this technique, the contrast enhancement in the pulmonary trunk is monitored and the scan is automatically initiated after a predefined threshold is reached. In clinical routine, the slice for bolus tracking in which the pulmonary trunk is visible, is manually localized by the radiographers in a scout view (also called topogram), which is an overview image acquired with low radiation exposure in anterior–posterior and/or lateral orientation. A suboptimal localization may cause the CT scan to be started at a suboptimal time, which affects the quality of the CT scan negatively and poses a risk to a correct diagnosis of pulmonary embolisms. If the CT scan has to be repeated because of poor image quality, the contrast agent must be administered again, further increasing the risk of contrast media induced nephropathy^9,10^. Manual localization of the pulmonary trunk in the scout view by the radiographer, apart from being time-consuming, is also subject to intra- and interindividual variability^11^.

An automation of this routine task could therefore lead to more standardized and optimized CTPA scans. Currently, automations of routine tasks are of growing interest, where Deep Learning based methods in particular have shown very good performance, often outperforming manually designed algorithms and sometimes reaching human levels^12,13^.

In this study, we investigate whether a deep neural network can be used for localizing the pulmonary trunk in CT scout views as well as radiographers.

## Methods

### Patients

Ethical approval for this retrospective study was granted by the local ethics committee (Ethik-Kommission, Medizinische Fakultät der Universität Duisburg-Essen, Germany; 29-9466-BO), and informed consent was waived. All procedures were performed in accordance with the relevant guidelines and regulations.

Based on a query in our picture archiving and communication system (PACS), 750 studies of patients who have received a thorax CT between March 2003 and February 2020 were randomly chosen and anonymized. This dataset was manually checked for study inclusion based on the following in- and exclusion criteria: Inclusion criteria were the presence of a corresponding scout view obtained in anterior–posterior projection. Exclusion criteria were age < 18 years, incomplete scout view which did not show the entire chest, scout views with a pixel spacing not equal to 1.0 mm, reconstructed CT slice thickness larger than 5 mm, severe anatomical deviations such as distinct thoracic deformity or pneumectomy or congenital heart defects. Based on these criteria, 620 scans from 563 patients were included in the study.

**Figure 1:**
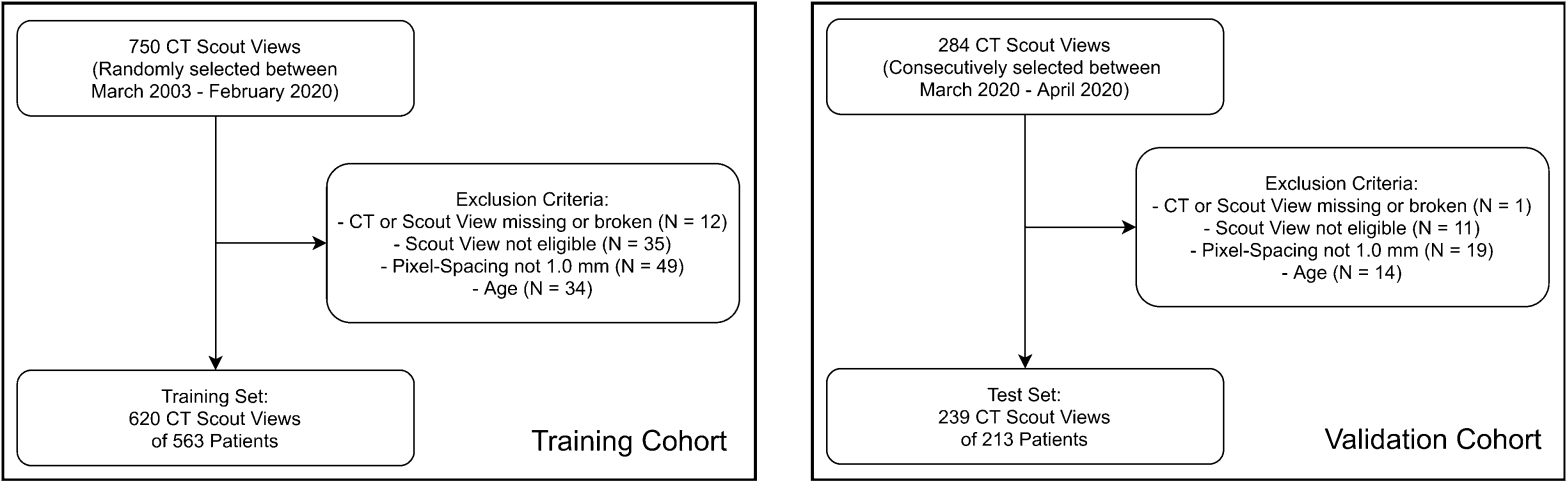
Patient flowchart for the training and the validation cohort with inclusion and exclusion criteria.

For evaluation of the trained neural network, a second, consecutive data set with patients from 1. March 2020 until 15. April 2020 was created with the same inclusion and exclusion criteria. This resulted in a data set with 239 CT scans from 213 patients (Fig. 1).

### Scout view acquisition

The selected CT scans were performed on various Siemens CT scanners (Siemens Healthineers, Erlangen, Germany) (Supplementary Table S1). All scout views were acquired in inspiration in anterior–posterior direction with a tube voltage of 120 kV and tube currents between 20 and 100 mA (Supplementary Table S2).

### Annotations

**Figure 2:**
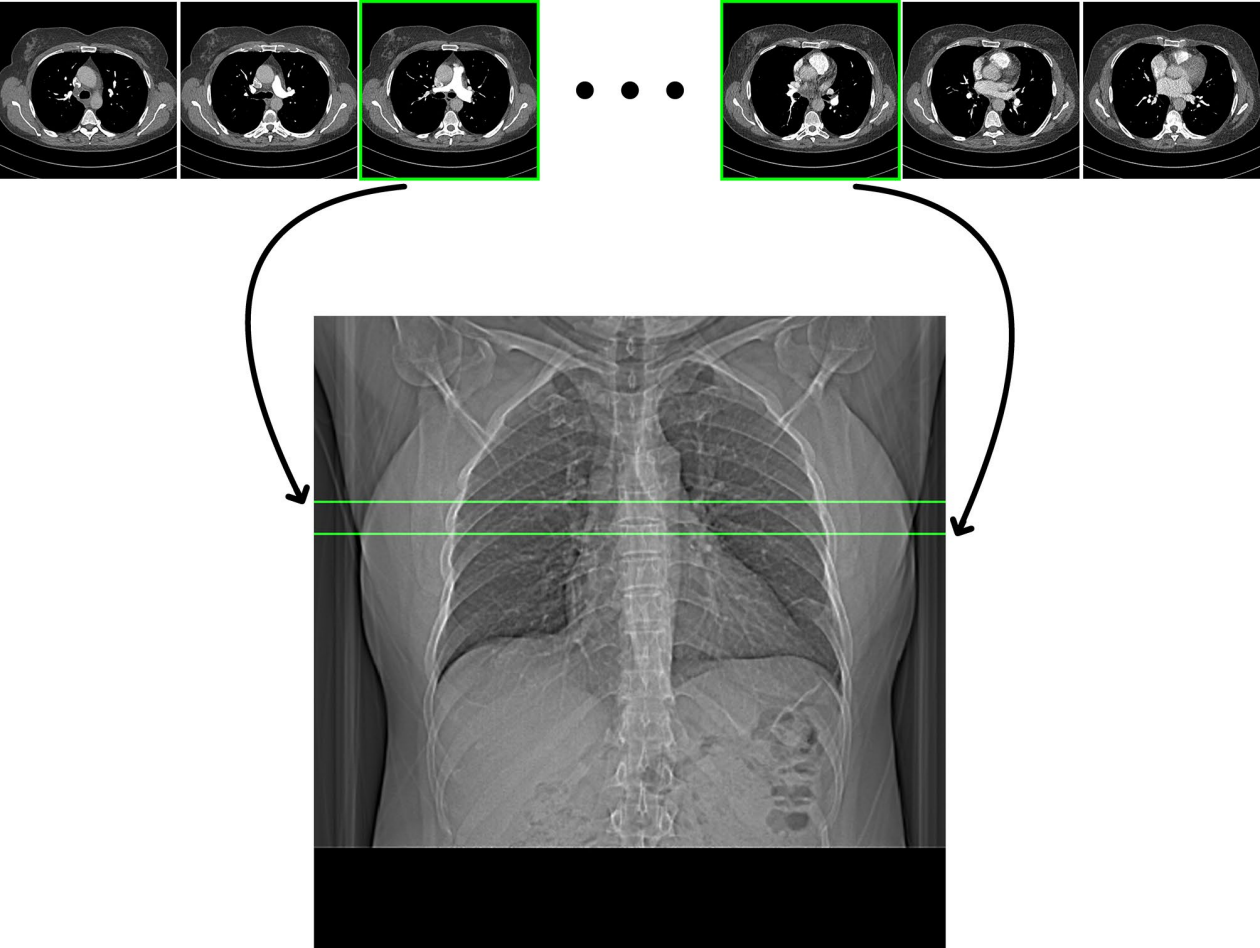
The axial slices of the CT scan between the cranial and caudal boundary of the pulmonary trunk were used to single out the 2D region in the CT scout view corresponding to the area of the pulmonary trunk (green). Images were cropped for better visibility.

Since an accurate localization of a single slice containing the pulmonary trunk in the CT scout view would exhibit very high intra- and inter-operator variability and subsequently impede the training of the neural networks, the CT scans were used to annotate the pulmonary trunk. Because the influence of different depths of inspiration between scout view and CT scan depends on the patient and cannot be accounted for directly, any slice between the lower (caudal) and the upper (cranial) boundary was considered to be correct, where the lower boundary was defined as the second slice above the pulmonary valve and the upper as the bifurcation of the pulmonary trunk into the right and left pulmonary artery (Fig. 2). Any slice in within these boundaries can therefore be used for bolus tracking. Depending on the patient, adjacent slices outside this region may also be useful, which is particularly the case at the upper boundary for anatomical reasons.

In addition, an optimal slice for bolus tracking, in which the pulmonary trunk was clearly visible, was defined as “reference standard”. Both annotations were performed by a resident and afterwards reviewed by a board-certified radiologist (K.N. with 16 years of experience). The annotation of all data (training and well as validation cohort) were performed before training and evaluation of the neural network.

Furthermore, using a custom-tailored software in Python, three radiographers (H.G.¸ A.S.Q. and S.B.) with experience between 3 and 10 years independently annotated all CT scout views in the test set by marking the slice position which they would have used for bolus tracking. This is in line with clinical routine, because there the radiographers do not annotate the full range, but only the slice position for the subsequent axial pre-scan.

### Neural network

Although a multitude of network architectures is currently in use for medical segmentation tasks^14^, their performance depends strongly on the data set and is not known beforehand. Nonetheless, the U-Net^15^ has proven to be a simple and very efficient architecture showing excellent results for medical segmentation tasks^16^ and was therefore chosen as network architecture. As the U-Net is able to generate arbitrary segmentations, but in the case at hand only a region (i.e. a simple bounding box with the same width as the CT scout view) has to be generated, the network was forced to produce bounding boxes by adding flattening layers to the output, which decreases the width of the output to one pixel (Text document, Supplementary Document 3).

As the learning rate is one of the key parameters during training, a fivefold cross-validation was used on the training set to tune this parameter^17^. Training was conducted using a fixed number of 50 epochs. Augmentations like vertical and horizontal shifts were applied to increase the sample size, but it was ensured that the augmentations would not invalidate the annotation. More details on the training of the neural network can be found online (Supplementary Document 3). The learning rate with highest average accuracy was then used to retrain a final model on all training data.

### Evaluation of the network

For evaluation, the retrained network was evaluated on the independent test set. This evaluation was done exactly once to avoid any bias by overfitting to the test set. The quality of the trained models was then evaluated in several ways:

Firstly, by determining whether at the chosen axial slice the pulmonary trunk was visible in the CT and therefore could have been used for triggering the subsequent CT scan. Here, the center position of the predicted region was chosen to be the slice in which the pulmonary trunk should be best visible in the corresponding CT.

Secondly, the accuracy of the prediction was defined by counting how often the selected slice is located in the region of the pulmonary trunk in the CT scout view. In addition, the distance of the chosen axial slice to the slice position which was defined as reference standard was measured. Since the CT scans were reconstructed with a slice thickness of 5 mm, a distance of less than about 2.5 mm would indicate that the network was able to select the best axial slice in all cases.

Thirdly, Intersection-over-Union (IoU) scores were computed, which measure the relative overlap of regions between the predicted region and the true region of the pulmonary trunk in the CT scout view. An IoU score below 0.5 would indicate that in the majority of cases the selected slice is not part of the region of the pulmonary trunk, while a higher IoU score would indicate that the neutral network was able to identify the region of the pulmonary trunk in the scout view.

Finally, since predictions of neural networks could potentially be unreasonable, for example the network could single out an area in the abdomen, the average distance to the nearest boundary was measured in case of an error (i.e. only in case of a prediction of a slice position outside the region of the pulmonary trunk).

### Comparison to the radiographers

The annotations of the three radiographers were similarly evaluated by the accuracy of the slice location, the distance to the optimal slice and the extend of error in case of a mistake. The annotations of each rater were then compared to the predictions of the neural network.

In addition, to directly compare the slice position generated by the network to those of the three radiographers, the corresponding four CT slices as well as the CT scout view (for overview purposes) were presented to the experienced radiologist (K.N.) in a blinded fashion. The radiologist then ranked the relative as well as absolute quality of the slices. For the relative quality, the CT slices were assigned a rank between 1 and 4 (where 1 indicates the best from the four presented CT slices and multiple CT slices can have equal ranks). For the absolute quality, the radiologist assessed whether the CT slices would be useful in clinical routine for bolus-tracking using three ranks (optimal, useful, useless). Although these two ratings are related, they are not directly comparable because, for example, the network may have selected a better CT slice than the radiographers, but that CT slice may still be useless for the subsequent bolus tracking.

### Sample size estimation and statistical analysis

A successful training of a neural network depends strongly on the nature of the problem and especially on the complexity and quality of the data. As such, there are currently no theoretical studies to determine a minimum sample for successful training, although first steps are taken in this direction^18^. For segmentation tasks, especially for the U-Net it has been demonstrated that excellent performance can be achieved with smaller sample sizes^15^. Since localization of the pulmonary trunk in the CT scout view can be regarded as a segmentation task and even more consists of a delimiting only a region instead of a full 2D segmentation, we considered a medium and manageable sample size of at least 500 to be sufficient for successful training. Accordingly, 750 CT scout views have been extracted to account for non-eligible ones.

For statistical testing, non-inferiority tests were used to verify the hypothesis that the neural network is not inferior to a radiographer. For comparing the accuracies, a non-inferiority test for paired binary data was used^19^. A sample size analysis with power of 0.8, a type I error rate of 5% and an accepted difference of 10% accuracy resulted in a minimum sample size of n = 172. For comparing distances, a sample size analysis of a non-inferiority (one-sided) t-test with power of 0.8 and an accepted difference of 5 mm (the slice thickness of the CT scan), an expected difference and a variance of 10 mm resulted in a minimum sample size of n = 52. Similarly, the ranking and the usefulness of the scan ranges of the radiographers were compared to those of the network by using a one-sided, Wilcoxon rank-sum test. A non-inferiority margin of 0.25 was taken for the mean rank as well as mean usefulness. A sample-size analysis with power 0.8, the minimum sample size was n = 137. Therefore, the sample size of the test set (N = 239) was deemed to be high enough.

Descriptive statistics were reported as mean ± standard deviation. A P-value < 0.05 was considered to indicate a statistically significant difference. No adjustments were performed for multiple testing. Statistical analyses were conducted using R 3.6.1.

## Results

The mean age of all patients was 59.9 ± 16.0 years (range 18 to 96 years), with 311 females and 465 males (Table 1). A difference between the age of the patients in the train set and the test set could be seen (P = 0.002). In the median, the area between the lower and upper boundaries comprised 5 CT slices (range 3–15), which corresponds to a length of 20 mm.

**Table 1:** Patient characteristics for the train and the test cohort.

	All (N = 776)	Train (N = 563)	Test (N = 213)	P
Female	311	220	91	0.40
Male	465	343	122	
Age (range)	59.9 (18–96)	58.7 (18–96)	63.1 (19–96)	0.002

The P-value denotes the significance of a chi-square and a t-test for sex and age respectively.

### Model evaluation

The cross-validation showed accuracy between 95.3 and 97.1% (Table 2) and the model with the highest accuracy was the one trained with a learning rate of 6e−5 (Supplemental Document 4). Therefore, this learning rate was fixed and a new (“final”) model was retrained on the whole training cohort and evaluated on the independent validation cohort. This model yielded an accuracy of 97.5%, which is on par with the accuracy seen during cross-validation (Table 2). On 6 out of 239 images the selected slice was outside the region of the pulmonary trunk, and the mean distance was 3.7 mm ± 1.0 mm. The distance to the optimal slice was 5.3 ± 4.2 mm, while the IoU score was 79.4 ± 14.3%.

**Table 2:** Accuracy of the radiographers.

	Accuracy [%] (errors/N)	P (rater vs U-Net)	Error distance (mean ± SD) [mm]	P (rater vs U-Net)	Distance to optimal slice (mean ± SD) [mm]	P (rater vs U-Net)	Mean Rank (rank distribution)	P (rater vs U-Net)	Mean usefulness (optimal/useful/useless)	P (rater vs U-Net)
Rater 1	83.3% (40/239)	< 0.001	7.6 ± 3.8	< 0.001	6.7 ± 5.6	< 0.001	1.5 (144/87/17/0)	< 0.001	0.69 (177/49/13)	< 0.001
Rater 2	49.0% (122/239)	< 0.001	11.4 ± 6.7	< 0.001	10.9 ± 8.1	< 0.001	**1.4** (167/50/20/2)	0.002	0.57 (165/46/28)	< 0.001
Rater 3	72.4% (66/239)	< 0.001	7.6 ± 4.9	< 0.001	12.9 ± 7.4	< 0.001	2.1 (79/76/71/13)	< 0.001	0.27 (101/101/37)	< 0.001
U-Net	**97.5%** (6/239)	–	**3.7 ± 1.0**	–	**5.3 ± 4.2**	–	1.5 (155/59/24/1)	–	**0.73** (179/56/4)	–

The mean rank and usefulness were judged by a board-certified radiologist. All P-values are measured between the corresponding rater and the U-Net. The mean usefulness indicates a mean score, where a score of 1.0 would be attained if all scan ranges were judged to be optimal, and − 1.0 if all scan ranges were useless. As all tests are for non-inferiority, a significant P-value indicates non-inferiority.

Best values were marked in bold.

### Comparison to radiographers

The accuracy of the raters (Table 2) was mixed (83.3%, 49.0% and 72.4%) and all lower than the accuracy of the network (97.5%). The non-inferiority test accordingly yielded indicated no inferiority of the synthetically generated scan ranges (P < 0.001 in all cases). With respect to the distance of the selected slice to the scan range of the pulmonary trunk in case of an error, the network again yielded the lowest error (3.7 ± 1.0 mm), and subsequently the non-inferiority test was statistically highly significant (P < 0.001 in all cases). This was also true for the distance to the reference slice in the CT scout view, where the network showed a mean distance of 5.3 ± 4.2 mm, again lower than all the distances of the raters (P < 0.001).

Considering the judgement of the experienced radiologist, rater 2 showed the best mean rank of 1.4, faring better than the neural network (mean rank 1.5). Using the non-inferiority margin of 0.25, again the neural network was non-inferior to all raters (P < 0.002 in all cases).

**Figure 3:**
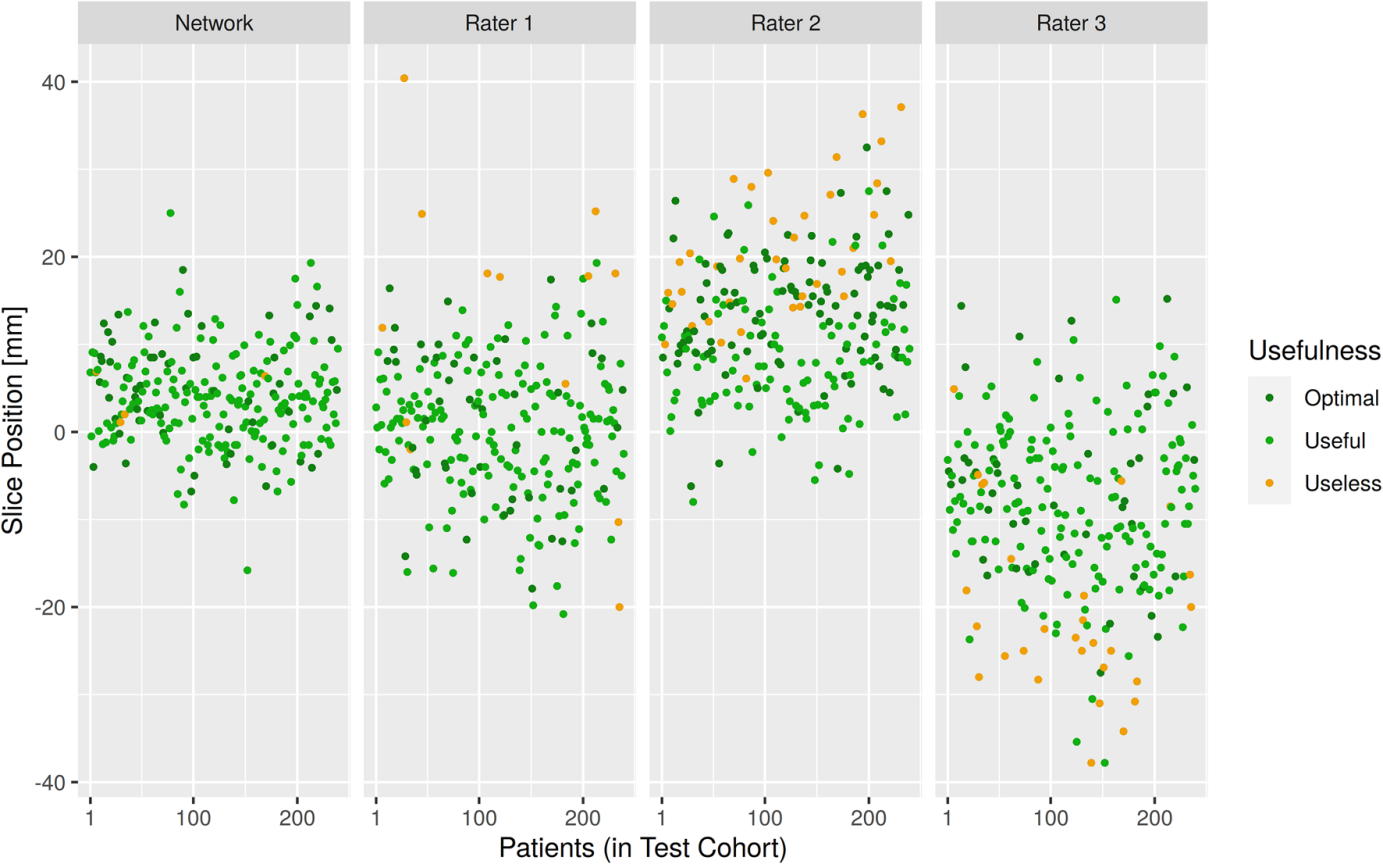
A visualization of the usefulness of the estimated slice positions of the pulmonary trunk of all radiographers and the predicted slice positions of the network on the test cohort. Slice positions are relative to the optimal slice position, which is different for each patient.

Also, the usefulness of the neural networks scan ranges were determined to be similarly useful to those of the radiographers (P < 0.001), moreover, all raters (including the neural network) were non-inferior to the others except for rater 3, who was inferior to all other raters (P = 1.0). A visualization of all predictions and their usefulness can be seen in Fig. 3.

## Discussion

The manual delimitation of anatomical structures in the scout view is a necessary and frequently used procedure when performing a CT scan. For CT angiograms of the pulmonary arteries (CTPA), a correct delimitation of the pulmonary trunk is necessary for correct bolus tracking and thus for optimal contrast enhancement in the subsequent CT scan. Since manual delimations in scout views are highly dependent on the experience of the radiographer, they exhibit a large intra- and inter-reader variability, which in turn has a significant impact on the quality of the subsequent CT scan. Therefore, Deep Learning was used in the present study for automatic delimitation of the pulmonary trunk in scout views.

Our study demonstrated that a neural network can delimitate the pulmonary trunk in scout views at least as well as an (experienced) radiographer. The slightly adapted U-Net yielded a very high overall accuracy of 97.5% in an independent test set, clearly higher than that of the best of the radiographers (83.3%). In case of a failure, which occurred in 6 out of 239 test scout views, the accuracy of the network did not suffer much as in mean the selected axial slice was very close to the region of the pulmonary trunk (3.7 ± 1.0 mm). In contrast, the best radiographer failed in 40 cases, and the mean distance was nearly twice as big (7.6 mm). Considering the distance to the reference slice, the network again fared better than the best radiographer (5.3 mm vs 6.7 mm). Thus, in terms of pure numbers, the network was able to outperform the radiographers, and its delimitations were thus significantly not inferior (P < 0.001).

A slightly different picture emerges when taking the judgment of the experienced radiologist into account. While statistical testing still significantly indicates the non-inferiority of the scan ranges of the network, rater 2 obtained a higher rank than the network (1.4 vs 1.5), while the usefulness of the scan ranges of rater 2 was lower (0.57 vs 0.73). This shows that even if the selected slice in the CT scout view seems perfect, possibly because of breathing artifacts, the corresponding slice in the CT scan is not necessarily optimal. The neural networks seemed to be able to take the breathing effects into account, as it is explicitly trained to relate the scan range in the CT scout view to the volume of the pulmonary trunk in the corresponding CT, whereas radiographers more likely rely to the landmarks they see in the CT scout view and are only able to account for breathing artifacts after longer experience.

Since the radiographers have different experience and work focus, a difference in performance was expected. Despite of this, it seems contradictory at first that the accuracy of the annotations of Rater 2 was lower than those of Rater 3, yet their usefulness was overall higher. This stems from the fact that Rater 2’s annotations are higher than necessary, while Rater 3’s annotations are slightly lower (Fig. 3). Due to the anatomy of the pulmonary trunk, slices at the upper boundary can often still be used for bolus tracking, while this is not so much the case at the lower boundary, which explains the apparent contradiction.

It is reasonable to expect that our network could be used in clinical routine, aiding especially less experienced radiographers. Integration of the network in clinical routine would be straight forward, as it could be performed directly on the CT scanner, as each prediction takes less than a few seconds (Supplemental Document 4). Even though in the independent validation cohort the results of the network were better than the best radiographer (179 vs 177 optimal slices), in 4 cases the prediction was not useable. Therefore, in clinical routine the network’s output should still be checked by the radiographer and, if necessary, corrected.

In contrast to our study, which aimed to define a single slice in the scout view in which the pulmonary trunk can be reliably detected, previous studies dealt exclusively with the detection of larger anatomical structures in the scout like the lung or the heart. In a study from Saalbach et al., bounding boxes around different anatomical structures were annotated directly in the CT scout view utilizing classical image processing methods^20^. In a similar recent study from Desphande et al. finer segmentations in the CT scout view were generated using deep learning techniques^21^. Besides the difference that in the previous studies only larger anatomical structures were detected and not a defined single slice, the corresponding CT scans were not considered in previous studies to evaluate the quality of the automatically generated annotation.

Our study introduced an adapted U-Net structure and showed that it can successfully locate the region of the pulmonary trunk in the CT scout view. From a machine learning perspective, this network seems to be unnecessary complex and not well optimized to the task. Initial experiments with a pretrained ResNet-18 network as well as a simple convolutional neural network to directly regress the slice position did not show excellent performance on the cross-validation folds. Since the U-Net showed better performance, possibly because it can use finer details for predictions due to its skip connections, it was selected for modelling. Nonetheless, it can be expected that a well-optimized regression network will achieve results similar or even better than the U-Net we presented. This topic should be carefully explored before clinical application.

Limitations apply to our study: Although multiple scanners were used, they all came from one vendor. Similarly, the independent validation cohort was collected from our own site. Thus, a study with multiple different CT vendors and a cohort from different sites should be conducted to verify the results of this study. Furthermore, although the U-Net showed very high performance, it is an off-the-shelf network and was only slightly modified to accommodate the structure of the data. More optimized training strategies^22^ or alternative, more sophisticated architectures, i.e. the Faster-RCNN^23^, could further increase accuracy.

In this work, an automated detection of the region corresponding to the pulmonary trunk in CT scout views was presented and demonstrated to be statistically non-inferior to three radiographers. As such, the network is able to select an axial slice in the corresponding CT that can be used for bolus triggering in a subsequent contrast-enhanced CT pulmonary angiogram.

## Supplementary Information

Supplementary Information 1.

Supplementary Information 2.

Supplementary Table S1.

Supplementary Table S2

## Data Availability

The datasets generated during and/or analyzed during the current study are available from the corresponding author upon reasonable request.
