# Supplementary material for: Detecting the pulmonary trunk in CT scout views using deep learning
Source: Sci Rep. 2021 May 13;11:10215. doi: 10.1038/s41598-021-89647-w (PMC8119439; doi:10.1038/s41598-021-89647-w)
Supplement: Supplementary file 1 — Supplementary Information 1. [file 41598_2021_89647_MOESM1_ESM.docx]

**Supplemental Digital Content 3**

***Preprocessing***

No scale normalization was applied as all CT scout views had a height and width of 512 pixel. As the left and right borders contained either empty space or uninteresting parts of the body, all images were cut symmetrically to a width of 384 pixels. Although similar black borders were mainly present at the bottom of the CT scout view, this was not consistently the case. Therefore, no cropping was performed at the lower or upper borders. Each CT scout view was intensity normalized by dividing by the maximum intensity value and multiplying by 255. Contrast-limited adaptive histogram equalization (CLAHE) was then applied with three different parameters (clipLimits of 16, 32, 64 with tile sizes of 1, 2 and 4 resp.) to increase contrast. The resulting three images were then concatenated, turning the grayscale CT scout view into a pseudo-colored RGB image.

***Neural Network architecture***

Although a multitude of network architectures is currently in use for medical segmentation tasks^1^, their performance depends strongly on the data set and is not known beforehand. Nonetheless, the U-Net^2^ has proven to be a simple and very efficient architecture showing excellent results for medical segmentation tasks^3^ and was therefore chosen as network architecture. In a nutshell, the U-Net consists of two parts: An analysis (encoder) part, which turns an image to a feature map, and a synthesis (decoder) part that turns a feature map back into an image. Both parts are connected via skip-connections, so that features computed during the analysis can be re-used in the synthesis (Figure S3.1).


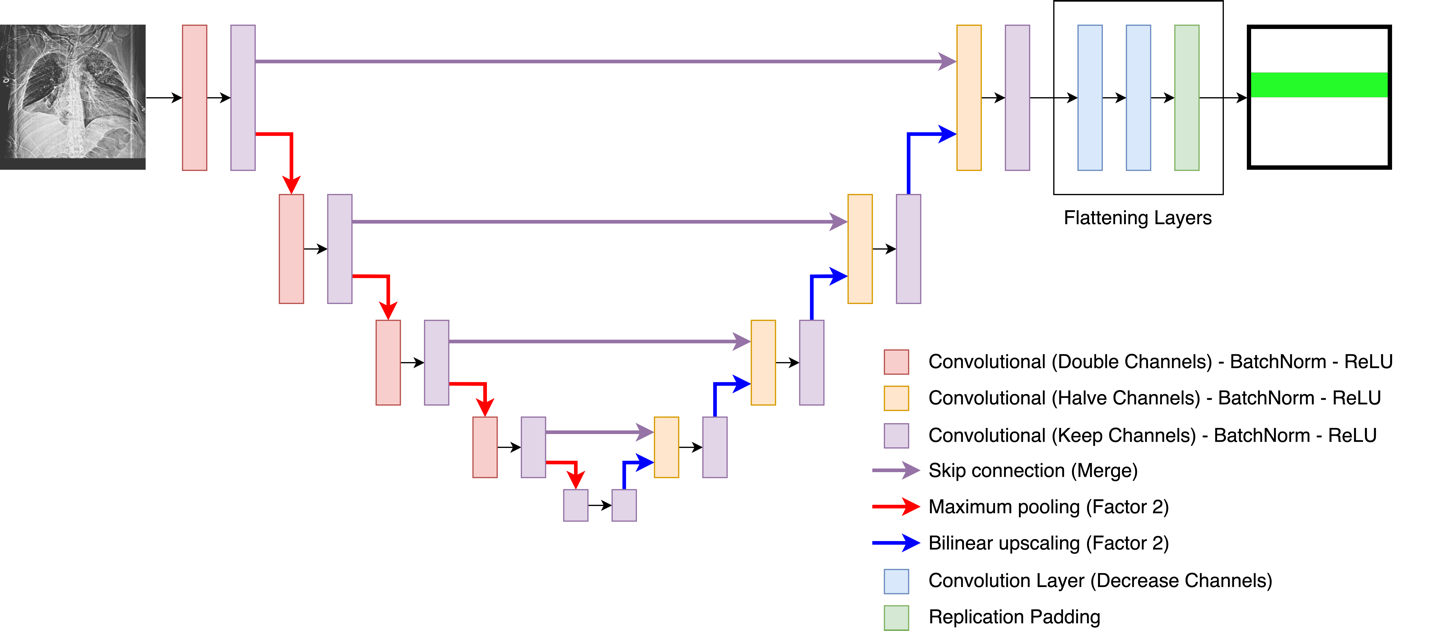


**Figure S3.1**: Schematic architecture of the U-Net. The input image is first convoluted with a kernel of size 3 and an output of 32 channels. It is then further processed by blocks of convolutional layers, batch norm layers and ReLu functions. The output is then further processed by flattening layers to ensure that the segmentation is a rectangular region. For more details to the U-Net refer to Ronneberger et al.^2^

Since a rectangular region (i.e. a simple bounding box with the same width as the CT scout view) has to be generated, but the U-Net is able to generate arbitrary segmentations, additional flattening layers were added to the output of the U-Net. The aim of these flattening layers is to force the neural networks to segment a rectangular region. The flattening layers consists of three layers (Figure S3.2): a convolution with a very wide kernel, a convolution with a smaller kernel, reducing the width of the predicted segmentation to a single pixel and a replication padding, which pads the segmentation back to its original shape by simple replication along the horizontal axis. This flattening can be seen as a compromise between several full convolutional layers with more common kernel widths (e.g. 3 or 5), which would introduce more parameters, and a global average pooling layer applied in the horizontal direction only, which would be free of parameters, but would possibly lack flexibility.


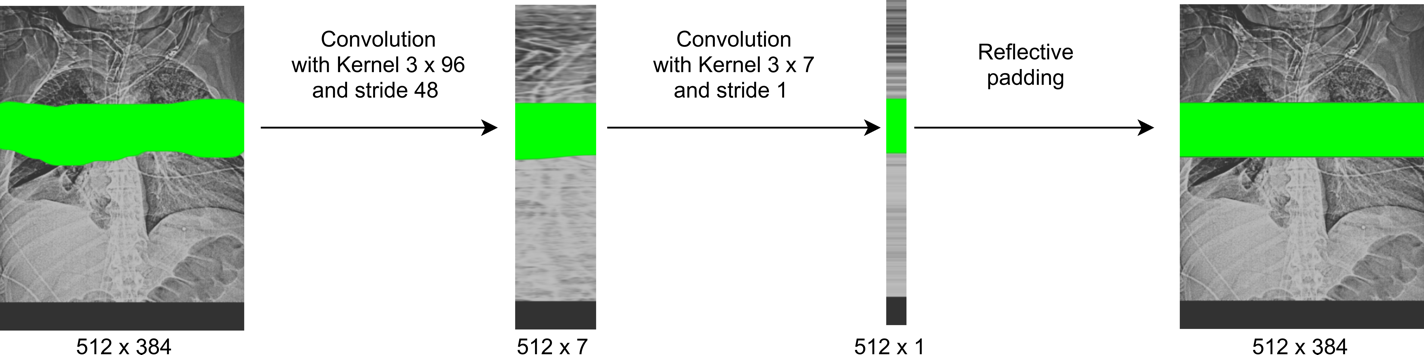


**Figure S3.2**: The flattening layer consists of two convolutional layers, so that the output has the same height and channels, but a width of only one pixel. First a convolution with very wide kernel (3x96 with a stride of 48) is used. This reduces the feature map to the size 512x7. A second convolution (with kernel size 3x7) is then used to reduce the feature map to 512x1. Using simple replication padding, the output is padded back to the original size of 512x384. This forces the output to be a rectangular region.

***Augmentations***

It is known that image transformations of training images, called augmentations, can increase the overall performance of neural networks as they work as additional regularization. This is especially important when training with data with smaller sample size. Therefore, several augmentations were applied: Uniform scaling (90% to 110%), horizontal and vertical shifts (2.5% of image width/height). Care was taken to avoid producing unrealistic CT scout views, e.g. no vertical or horizontal flip was applied. Additionally, it was ensured that used augmentations would not invalidate the annotation. For example, a very large rotation of the image would render the annotation useless as it would no longer be parallel to the imaging slices in the CT and thus the correct location of the pulmonary trunk could not be asserted. Thus, only small rotations (< 1.5 degrees) centered on the center of the region of the pulmonary trunk were applied. All augmentations except for the rotation were applied to both, the image and the annotation.

***Training***

A 5-fold cross-validation was used to estimate the performance of the network on the training set. For this, the 620 CT scout views were randomly divided patient-wise into 5 sets of equal size. Each set contained roughly 120 CT scout views (as a single patient could have multiple CT scout views, the count is not exactly 120), around 20% of the training data. In each round, one of the folds was set aside and only used for evaluation purposes after training, while the rest (around 500 samples) was used for training.

Training parameters were chosen as follows: The batch size B was fixed to 2, as very large batch sizes might lead to a “generalization gap”, i.e. a degradation of generalization performance.^4^ Weights were initialized using a normal distribution with standard deviation of 0.02. Training was conducted for 50 epochs. The squared mean loss (L2 loss) was used. Following Heusel et al,^5^ the learning rate is one of the key parameters in training, and so the learning rate was the only parameter tuned during cross-validation and was chosen among [0.0006, 0.0003, 0.0001, 0.00006, 0.0003, 0.00001]. During training, the learning rate was kept fixed for 25 epochs, before it was linearly scaled down to 0 in the next 25 epochs.

As the network produces a rectangular region, but a single slice needs to be predicted, the center position of the predicted region was chosen to be the slice in which the pulmonary trunk should be best visible in the corresponding CT.

Accordingly, the accuracy of the network was defined by counting how often the selected slice is located in the region of the pulmonary trunk in the CT scout view. The model with highest average accuracy over the cross-validation folds was then selected as the best model and was subsequently retrained with all training data.

***Constant predictor***

In general, it can happen that a neural network makes a constant prediction and therefore has no added value. To check this, a constant predictor was created as follows: The average scan range was calculated over all annotations in the test set. The constant predictor then predicted the middle of this scan range for each image. The performance of this model was then compared to the trained neural network.

***Software***

For training and evaluation of the network, Python 3.6 and pytorch 1.4 were used. Augmentations were performed with the torchvision and OpenCV libraries. All networks were trained on commodity hardware (AMD Ryzen Threadripper 2950X with 128GB of RAM, an 1 TB M2.SSD drive and a NVidia TITAN RTX card, running Ubuntu 18.04 LTS). The source code of the experiments will be published on the open source in a repository on github [https://github.com/aydindemircioglu/pulmonary.trunk].

***References***

1. Simpson AL, Antonelli M, Bakas S, et al. A large annotated medical image dataset for the development and evaluation of segmentation algorithms. *arXiv:1902.09063 [cs, eess]*. 2019. Available at: http://arxiv.org/abs/1902.09063. Accessed December 13, 2019.

2. Ronneberger O, Fischer P, Brox T. U-Net: Convolutional Networks for Biomedical Image Segmentation. *arXiv:1505.04597 [cs]*. 2015. Available at: http://arxiv.org/abs/1505.04597. Accessed January 17, 2019.

3. Isensee F, Petersen J, Kohl SAA, et al. nnU-Net: Breaking the Spell on Successful Medical Image Segmentation. *arXiv:1904.08128 [cs]*. 2019. Available at: http://arxiv.org/abs/1904.08128. Accessed April 21, 2019.

4. Hoffer E, Hubara I, Soudry D. Train longer, generalize better: closing the generalization gap in large batch training of neural networks. In: Guyon I, Luxburg UV, Bengio S, et al., eds. *Advances in Neural Information Processing Systems 30*. Curran Associates, Inc.; 2017:1731–1741. Available at: http://papers.nips.cc/paper/6770-train-longer-generalize-better-closing-the-generalization-gap-in-large-batch-training-of-neural-networks.pdf. Accessed July 20, 2020.

5. Heusel M, Ramsauer H, Unterthiner T, et al. GANs Trained by a Two Time-Scale Update Rule Converge to a Local Nash Equilibrium. In: Guyon I, Luxburg UV, Bengio S, et al., eds. *Advances in Neural Information Processing Systems 30*. Curran Associates, Inc.; 2017:6626–6637. Available at: http://papers.nips.cc/paper/7240-gans-trained-by-a-two-time-scale-update-rule-converge-to-a-local-nash-equilibrium.pdf. Accessed May 28, 2020.
