# Supplementary material for: Detecting the pulmonary trunk in CT scout views using deep learning
Source: Sci Rep. 2021 May 13;11:10215. doi: 10.1038/s41598-021-89647-w (PMC8119439; doi:10.1038/s41598-021-89647-w)
Supplement: Supplementary file 2 — Supplementary Information 2. [file 41598_2021_89647_MOESM2_ESM.docx]

**Supplemental Digital Content 4**

***Results of the Cross-Validation***

The cross-validation showed accuracies between 95.3 and 97.1% (Table S4). In cases of an error, i.e. if the network selected a slice outside the region of the pulmonary trunk, the mean distance between selected slice and the region of the pulmonary trunk were between 6.8 and 14.3 mm. The average distance of the selected slice to the optimal slice did not vary much and was between 5.3 and 5.8 mm. Similarly, the dice scores were not much dependent on the learning rate and were between 77.6 and 78.5%.

| **Learning Rate** | **Accuracy [%] (Errors/N)** | **Error Distance (Mean ± SD) [mm]** | **Distance to Optimal Slice (Mean ± SD) [mm]** | **IoU Score (Mean ± SD) [%]** |
| --- | --- | --- | --- | --- |
| 0.0006 | 95.3 (29/620) | 7.8 ± 7.1 | 5.5 ± 5.1 | 64.8 ± 19.1 |
| 0.0003 | 96.0 (25/620) | 6.8 ± 8.3 | 5.4 ± 4.7 | 67.5 ± 18.1 |
| 0.0001 | 95.6 (27/620) | 8.8 ± 10.4 | 5.4 ± 5.0 | 66.0 ± 19.6 |
| 0.00006 | 97.1 (18/620) | 8.9 ± 13.9 | 5.3 ± 4.9 | 65.5 ± 18.9 |
| 0.00003 | 96.0 (25/620) | 14.3 ± 23.3 | 5.8 ± 6.9 | 65.5 ± 19.3 |
| 0.00001 | 96.8 (70/620) | 12.3 ± 12.5 | 5.6 ± 5.5 | 66.2 ± 19.2 |

**Table S4**: Cross-validation results of the best models for each model architecture.

***Timings***

The prediction of a scan range for a single CT scout view took around 0.3 seconds on average (including all I/O times) on the NVidia Titan RTX. If only a single CPU core is used (on an AMD Threadripper 2950X), as might be necessary in clinical routine, prediction of a scan range took around 4.5 seconds on average (including all I/O times).

***Constant Predictor***

The average scan range covered scan lines 135-156 (in pixels, with respect to the CT scout views with pixel height of 512) and had a mean size of 21 pixel. Predicting the central slice of this area (corresponding to the scan line 145), the constant predictor showed an accuracy of 38.1% (148/239 errors), a mean error difference of 25.5 +/- 27.0 mm, a distance to the optimal slice of 24.2 +/- 25.8 mm and an IoU score of 23.4 +/- 28.1. As the trained neural network showed much better performance, it can be concluded that the network does not simply predict a fixed scan range.
