# Supplementary material for: Detecting the pulmonary trunk in CT scout views using deep learning
Source: Sci Rep. 2021 May 13;11:10215. doi: 10.1038/s41598-021-89647-w (PMC8119439; doi:10.1038/s41598-021-89647-w)
Supplement: Supplementary file 3 — Supplementary Table S1. [file 41598_2021_89647_MOESM3_ESM.docx]

**Supplemental Digital Content 1**

| **Scanner** | **Detector Collimation** | **Reconstruction Kernel** | **Peak Tube voltage** |
| --- | --- | --- | --- |
| Siemens Somatom Force | 96 x 0.6 mm | Br40d | 80-150 kVp |
| Siemens Somatom Definition AS+ | 64 x 0.6 mm | I30f | 100-120 kVp |
| Siemens Somatom Definition Flash | 64 x 0.6 mm | I30f | 100-120 kVp |
| Siemens Volume Zoom | 4 x 1.0 mm | B30f | 120-140 kVp |
| Siemens Sensation 16 | 16 x 0.75 mm | B30f | 120 kVp |

**Table**: CT scanner and scan parameters used for the acquisition of the CT scout views. The CT scan was acquired in supine position, head first and in deep inspiration. Image reconstructions were performed using filtered back projection.
