# Supplementary material for: Detecting the pulmonary trunk in CT scout views using deep learning
Source: Sci Rep. 2021 May 13;11:10215. doi: 10.1038/s41598-021-89647-w (PMC8119439; doi:10.1038/s41598-021-89647-w)
Supplement: Supplementary file 4 — Supplementary Table S2 [file 41598_2021_89647_MOESM4_ESM.docx]

**Supplemental Digital Content 2**

| **Scanner** | **Total scout views (N = 859)** | **Scout views in training cohort (N = 620)** | **Scout views in validation cohort (N = 239)** | **Tube voltage** | **Tube current** |
| --- | --- | --- | --- | --- | --- |
| Siemens Somatom Force | 316 | 149 | 167 | 120 kV | 20 mA |
| Siemens Somatom Definition AS+ | 218 | 179 | 39 | 120 kV | 35 mA |
| Siemens Somatom Definition Flash | 158 | 126 | 32 | 120 kV | 35 mA |
| Siemens Volume Zoom | 107 | 107 | 0 | 120 kV | 100 mA |
| Siemens Sensation 16 | 60 | 59 | 1 | 120 kV | 50 mA |

**Table**: CT scanners and number of CT scout views acquired.
